# Effects of Lactobacillus Plantarum and Pullulanase Modification on the Physicochemical Properties and Functional Characteristics of Highland Barley Flour

**DOI:** 10.3390/foods15162771

**Published:** 2026-08-07

**Authors:** Xizong Gu, Tianying Sun, Wan Wang, Chunli Song, Jian Ren, Jinzhao Xu

**Affiliations:** 1 College of Food and Bioengineering, Qiqihar University, Qiqihar 161006, China; 2 Engineering Research Center of Plant Food Processing Technology, Ministry of Education, Qiqihar 161006, China

**Keywords:** combined modification, starch, physicochemical properties, biomodification, soluble dietary fiber

## Abstract

Due to its high amylopectin content and absence of gluten proteins, highland barley flour exhibits poor dough-forming capacity and processability. This study investigated combined modification strategies, Lactobacillus plantarum fermentation and pullulanase treatment, to improve the flour’s physicochemical and processing characteristics. Results indicated that these treatments significantly altered the morphological appearance and nutritional composition of highland barley flour, as well as increasing the amylose content. The group subjected to enzymatic hydrolysis followed by fermentation achieved the highest amylose content of 29.09%, while the amylopectin content decreased to 39.99%. Meanwhile, the content of soluble dietary fiber rose from 18.32% to 60.64%. Structural analysis revealed that enzymatic hydrolysis and fermentation could disrupt the hydrogen bond network and double-helix structure of starch, thereby reducing gelatinization parameters, with the most pronounced effect observed in the combined treatment of prior enzymatic hydrolysis followed by fermentation. For processing properties, this combination markedly improved the water-holding capacity and reduced the oil-holding capacity of the flour. Additionally, this combined modification exerted the most prominent enhancement on dough springiness, chewiness, viscoelasticity and rheological properties. Consequently, the combined modification of enzymatic hydrolysis and fermentation is a promising strategy to improve the nutritional value and processability of highland barley flour.

## 1. Introduction

Highland barley (*Hordeum vulgare* L. var. Nudum Hook. f.) is an important staple crop in plateau regions. It can grow stably at elevations of approximately 4000 m and offers advantages such as strong cold tolerance, early maturity, a short growing season, broad adaptability, and relatively high yields [1]. In addition to serving as a staple food, highland barley also possesses significant economic and nutritional value. Studies have shown that it is rich in dietary fiber and is considered a “green”, safe, and health-promoting grain resource [2]. Compared to barley and wheat, the content of major nutrients in highland barley fluctuates more significantly: starch ranges from approximately 47.90% to 79.0%, protein from approximately 6.35% to 21.00%, and β-glucan ranges from approximately 3.66% to 8.00%, with β-glucan levels typically being higher [3,4]. In recent years, it has also been discovered that highland barley possesses potential bioactive properties, including anticancer effects as well as lipid-lowering and blood-glucose-lowering effects, which may help prevent diabetes, hypertension, and cardiovascular diseases [5]. However, the primary carbohydrate in highland barley flour is starch (accounting for approximately 47.90–79.0% of the grain) [1], and its relatively dense structure often leads to insufficient hydration capacity, poor flavor, and limited dough processing properties [6], thereby restricting its application in the modern food industry to some extent [7]. Consequently, regulating the structural and functional properties of highland barley flour through appropriate modification techniques to enhance processing suitability and nutritional value has become a research focus [8].

Currently, common methods for modifying highland barley flour include physical, chemical, and biological approaches [9,10]. Among these, after extrusion and puffing treatment, the molecular weight of highland barley starch decreases, the ratio of amylose to amylopectin increases, and swelling capacity and viscosity are enhanced [6]; however, studies indicate that this treatment reduces total polyphenol and free polyphenol content by 52.7% and 89.3%, respectively, leading to a significant decline in antioxidant activity. Wet heat treatment improves the gelatinization stability of highland barley flour, increasing the β-glucan content from 5.60% to 6.76%; however, the gelatinization enthalpy increases from 4.99 J/g to 5.55 J/g, indicating increased energy consumption during the starch molecular rearrangement process [11]. Chemical modification, represented by starch derivatization techniques such as acetylation, can alter the molecular structure, gelatinization, and rheological properties of highland barley starch; however, the risk of chemical residues and clean-label constraints on product labeling limit its widespread application in the field of natural food ingredients [12].

Meanwhile, microbial fermentation and enzymatic hydrolysis have been extensively studied for their mild conditions, high specificity, and safety. Fermentation is an effective method for modifying highland barley with biological enzymes. During fermentation, microorganisms consume carbohydrates and produce various metabolites, such as acids and gases, thereby altering the starch [13]. Enzymatic treatment is generally used to hydrolyze polysaccharides in grains, fruits, and vegetables while imparting other functional properties to foods. In recent years, the application of enzyme preparations in food has become a research hotspot. Among these, the most commonly used enzyme is pullulanase, which has been the subject of numerous studies regarding its ability to reduce food digestibility. For example, pullulanase shows great potential for producing resistant starch; studies have found it to be more effective than other reported debranching enzymes in producing resistant starch from peas. These studies collectively demonstrate that enzymatically modified foods hold immense potential for regulating digestibility and nutritional functionality [14]. Despite prior investigations into the modification of highland barley flour via microbial fermentation or enzymatic hydrolysis alone, the sequential effects of combined microbial and enzymatic modification, specifically the “fermentation first then enzymatic hydrolysis” versus “enzymatic hydrolysis first then fermentation” regimes, remain unexplored. These two sequences are governed by distinct mechanisms: in the enzyme-first combined process, the generation of short-chain amylose glucans may alter the carbon-source composition, thereby influencing microbial acid production; conversely, in the fermentation-first combined process, pH fluctuations and substrate pre-degradation may modulate the efficiency of subsequent pullulanase action [15]. Such temporal differences in turn drive the rearrangement of the starch β-glucan–protein interaction network, leading to non-additive changes in dietary fiber solubilization, starch crystallite packing, and dough hydration capacity [16]. To date, a systematic comparison of the structural evolution and functional outcomes under these two sequential combined-modification strategies is lacking. Nevertheless, this combined approach holds promise for achieving integrated effects unattainable by either single treatment alone, particularly in enhancing soluble dietary fiber content, improving starch physicochemical properties, and tailoring dough rheological behavior, thereby offering significant application potential [17].

In this study, highland barley flour served as the raw material, and four modification methods were employed: Lactobacillus plantarum fermentation, pullulanase hydrolysis, combined modification with fermentation first, and combined modification with enzymatic hydrolysis first. The study systematically investigated how these treatments affected color, microstructure, particle size distribution, starch composition, soluble dietary fiber content, thermal properties, X-ray diffraction, water and oil retention capacity, and dough texture and rheological properties. The aim was to elucidate the regulatory mechanisms by which different modification methods influence the structure and functionality of highland barley flour, thereby providing a theoretical basis and technical support for developing high-value-added functional highland barley ingredients and improving their processing adaptability.

## 2. Materials and Methods

### 2.1. Materials

Highland barley was obtained from Tibet Jixiang Grain Agriculture Development Co., Ltd. (Lhasa, Tibet, China). Direct-plated Lactobacillus plantarum (live cultures; 1.0 × 10^10^ CFU/g) was purchased from Shanxi Taizhi Biotechnology Co., Ltd. (Xi’an, Shaanxi, China). Pullulanase (food-grade; activity 1.0 × 10^5^ U/g) was obtained from Guangdong Guangtai Food Technology Co., Ltd. (Dongguan, Guangdong, China). Petroleum ether (analytical reagent) was purchased from Liaoning Quanrui Reagent Co., Ltd. (Jinzhou, Liaoning, China). Kits for determining amylopectin content and amylose content were obtained from Beijing Bokesi Biotechnology Co., Ltd. (Beijing, China). Other chemicals were of analytical grade.

### 2.2. Modification by Lactobacillus Plantarum Fermentation

After cleaning the highland barley, it was dried in an oven at 50 °C for 12 h, ground with a grinder, sieved through a 100-mesh sieve, and stored in a dry, sealed container.

Based on relevant literature, 30 g of highland barley flour was added to a beaker, 60 mL of distilled water was added, and the pH was adjusted to 4. In a sterile laminar flow cabinet, 1.6% (*w*/*v*) direct-inoculated Lactobacillus plantarum was added (based on dry weight) and stirred thoroughly for 5 min to ensure uniform distribution of the bacteria. Then the mixture was sealed and fermented in a water-jacketed incubator at 37 °C for 30 h [18]. After fermentation, it was centrifuged at 4000 r/min (2000× *g*) for 15 min. Subsequently, the highland barley flour was dried in a 50 °C oven for 12 h, ground, sieved through a 100-mesh sieve to obtain fermented highland barley flour, and stored for testing.

### 2.3. Modification by Pullulanase Enzymatic Hydrolysis

In accordance with relevant literature, 30 g of highland barley flour was added to a beaker, 60 mL of distilled water was added, and the pH was adjusted to 5. Then 0.06% (*w*/*w*, based on dry weight) of pullulanase was added. The reaction mixture was placed in a magnetic stirrer with heating and incubated in a water bath at 45 °C and 200 r/min for 60 min to perform enzymatic hydrolysis [19]. After the reaction, it was centrifuged at 4000 r/min (2000× *g*) for 15 min. The hydrolyzed highland barley flour was dried in a 50 °C oven for 12 h, ground, and sieved through a 100-mesh sieve to obtain enzymatically hydrolyzed highland barley flour and stored for testing.

### 2.4. Combined Modification

#### 2.4.1. Fermentation Before Enzymatic Hydrolysis

Lactobacillus plantarum was fermented at a starter concentration of 1.6% (*w*/*w*) and a constant temperature of 37 °C for 30 h. After fermentation, the pH was adjusted to 5, and 0.06% (*w*/*w*) pullulanase was added. Enzymatic hydrolysis was carried out by stirring the mixture in a water bath at 45 °C for 60 min [17]. Subsequent procedures were the same as those described in Section 2.1.

#### 2.4.2. Enzymatic Hydrolysis Before Fermentation Modification

First, 0.06% (*w*/*w*) pullulanase was added and the mixture was stirred in a 45 °C water bath for 60 min to perform enzymatic hydrolysis modification. After modification, the pH was adjusted to 4, then Lactobacillus plantarum fermentation was performed using a 1.6% (*w*/*w*) inoculum at 37 °C for 30 h [20]. Subsequent procedures are the same as those described in Section 2.1.

### 2.5. Chemical Analysis of Highland Barley Flour and Color Measurement

The chemical composition of highland barley flour was determined according to standard methods: moisture content was determined by AACC 45-15.02; total fat content was determined by Soxhlet method AACC 30-23; protein content was determined by Kjeldahl method AACC 46-12, with a nitrogen-to-protein conversion factor of 6.25; ash content was determined by AACC 08-01; sugar content was determined by Luff–Schoorl method AACC 80-68. Total starch was measured using an enzymatic kit (Total Starch Content Test Kit, Beijing Bokesci Biotechnology Co., Ltd., Beijing, China).

The color of highland barley flour samples was measured using a colorimeter (CR-10 Plus, Hangzhou Keshengxing Instruments Co., Ltd., Hangzhou, China). Based on this, whiteness (W) is calculated using the formula to comprehensively evaluate the sample’s whiteness. Whiteness is calculated using Equation (1):

(1) $$W=100-\sqrt{(100-L{)}^{2}+a^{2}+b^{2}}$$

### 2.6. Scanning Electron Microscopy (SEM)

SEM observation was performed according to a published protocol [21] with slight modifications. Samples of different modified highland barley flour powders were spread evenly and firmly attached to the instrument stage using double-sided conductive tape, ensuring uniform distribution of the sample particles. To improve the conductivity of the sample surface, the sample surface was coated with a gold film of approximately 10~20 nm thickness using an ion sputter coater (MC1000, Hitachi High-Technologies Corporation, Tokyo, Japan). The sample was transferred to the SEM (Thermo Fisher Helios 5 CX, Thermo Scientific, Waltham, MA, USA) observation chamber, the electron gun accelerating voltage was adjusted to 10 kV, and the surface microstructure of the different modified highland barley flour particles was observed.

### 2.7. Particle Size Analysis

With reference to the method of Namazifar et al. [12], with slight modifications, 0.1 g of highland barley flour subjected to different modification methods was taken, diluted 100-fold, and ultrasonicated at 200 W for 5 min to achieve a uniform dispersion of the sample solution. The instrument parameters were set as follows: absorption rate 0.01, temperature 25 °C, dispersant refractive index (RI) 1.330, and measurement duration 60 s. The particle size distribution of the samples was determined using a Malvern particle size analyzer.

### 2.8. Determination of Amylopectin and Amylose Contents

Amylopectin and amylose contents were measured using commercial assay kits: an amylopectin content detection kit (Beijing Bokesi Biotechnology Co., Ltd., Beijing, China) and an amylose content detection kit (Beijing Bokesi Biotechnology Co., Ltd., Beijing, China), respectively. The amylopectin content and amylose content in dried highland barley flour (dry basis) were determined according to the manufacturer’s instructions.

### 2.9. Determination of the Effect on Soluble Dietary Fiber Content

Referring to the method of Sharm et al. [22] with slight modifications, 1 g of the sample to be tested was extracted in a water bath at a sample-to-liquid ratio of 1:20 (*m*/*v*). After adjusting the pH of the extract to 6.0, thermostable α-amylase (1000 U/g) was added and digested at 95 °C for 35 min. When the temperature dropped to 55 °C, the pH was adjusted to 9.5, and alkaline protease (800 U/g) was added for digestion for 30 min. The pH was then adjusted to 4.5, and glucoamylase (1000 U/g) was added for digestion for 30 min. After the reaction was completed, the enzyme was inactivated in a boiling water bath for 10 min, followed by centrifugation at 8000 r/min (6600× *g*) for 15 min. The supernatant was collected, mixed with 4 volumes of 95% (*v*/*v*) ethanol, and precipitated at 4 °C overnight. The mixture was centrifuged at 8000 r/min (6600× *g*) for 15 min, and the precipitate was redissolved, concentrated until free of ethanol odor, and then freeze-dried and weighed. The soluble dietary fiber content is calculated using Equation (2):

(2) *SDF* (%) = *m*_1_/*m*_2_

where: m_1_ is the mass of the obtained SDF; m_2_ is the sample mass.

### 2.10. Fourier Transform Infrared Spectroscopy

With slight modifications based on the literature [23], 1 mg of modified highland barley flour was weighed and mixed with 100 mg of potassium bromide to prepare a sample. A full-wave scan from 4000 to 400 cm^−1^ was performed using FTIR (PE, Shelton, CT, USA) to analyze the changes in major absorption peaks and, in conjunction with an infrared spectral library, to analyze changes in molecular structure.

### 2.11. X-Ray Diffraction Analysis

Relative crystallinity was determined using an X-ray diffractometer (Bruker-AXS, Karlsruhe, Germany). An appropriate amount of sample was placed in the instrument for X-ray analysis. The experimental conditions were set as follows: the 2θ scan range was set to 5–50°, and the scan speed was set to 2°/min.

### 2.12. Differential Scanning Calorimetry (DSC) Measurement

Thermal properties were measured using a differential scanning calorimeter, including onset temperature (T*_o_*), peak temperature (T*_p_*), and gelatinization enthalpy (Δ*H*). Following the method of Gan et al. [14], the differential scanning calorimeter test conditions were set as follows: 4.0 mg of different modified highland barley flour samples were placed in aluminum pans, and 8.0 μL of deionized water was added, then sealed. Before testing, the aluminum pans were equilibrated overnight in a 4 °C refrigerator. During testing, the temperature was increased from 30 °C to 95 °C at a rate of 10 °C/min, using an empty aluminum pan as a reference to calibrate the DSC instrument (Mettler Toledo Instruments, Shanghai, China).

### 2.13. Determination of Water- and Oil-Holding Capacity

Adapted from Joshi et al. [24] with appropriate modifications: approximately 1 g of the sample to be tested (m_1_) was placed in a clean, dry 50 mL centrifuge tube, and 40 mL of distilled water was added and stirred with a glass rod to ensure thorough dispersion and suspension. It was centrifuged at 6000 rpm for 16 min, then the supernatant was discarded. The centrifuge tube was inverted and left to stand for approximately 2 min. Once no visible droplets remained, the total mass of the centrifuge tube and the wet precipitate (m_3_) was weighed. The determination was repeated three times and the arithmetic mean was taken as the final result.

The method for determining oil-holding capacity is the same as that for water-holding capacity, except that distilled water is replaced with vegetable oil during the test. Water retention was determined according to Equation (3):

(3) Water-Holding Capacity (%) = (m_3_ − m_2_)/m_1_

where: m_1_ is the mass of the sample taken; m_2_ is the mass of the empty centrifuge tube; m_3_ is the mass of the centrifuge tube with sediment.

### 2.14. Dough Preparation

#### 2.14.1. Determination of Dough Textural Properties

The textural properties of the dough samples were characterized using a slightly modified version of a previously described method [25]. Different barley flours were prepared into doughs with the same specifications, using a flour-to-water ratio of 5:4 (g/mL), and tested using a texture analyzer (TMS-PR, Beijing Ying Sheng Hengtai Technology Co., Ltd., Beijing, China). A 75 mm diameter disc compression probe was selected, with a range of 100 N, a test speed of 60 mm/min, an interval of 5 s between two compressions, 50% deformation, and a trigger force of 0.1 N as the test conditions for elasticity measurement.

#### 2.14.2. Dough Rheological Properties Testing

Following Zong et al. [26] with slight modifications, a rheometer (Kinexus, Malvern Company, Malvern, UK) was used to measure the dynamic viscoelasticity of the dough. Barley flour with different modifications was prepared into dough of the same specifications at a flour-to-water ratio of 5:4 (g/mL), balanced at 25 °C for 30 min, and then transferred to a parallel plate. The dough was measured on a 50 mm diameter plate with a 1 mm gap. After performing a strain sweep test (0.01–100%), a frequency sweep was conducted at a strain rate of 0.1%, with a frequency range from 1 Hz to 10 Hz. The elastic modulus (G′), viscous modulus (G″), and loss tangent tanδ (tanδ = G″/G′) of the differently modified barley flours were recorded.

### 2.15. Statistical Analysis

All experiments were repeated at least three times (*n* = 3), all analytical measurements were performed in triplicate, and the average value was used as the representative data point for that replicate. Data are presented as mean ± standard deviation (*n* = 3) and were analyzed using analysis of variance (ANOVA), followed by Tukey’s test (*p* < 0.05), using SPSS version 19.0 statistical software (SPSS Inc., Chicago, IL, USA). The charts were created using Origin 8.5 software (Origin Labs, Northampton, MA, USA).

## 3. Results and Discussion

### 3.1. Results of Basic Components and Color Measurement

As shown in Table 1, compared with the original powder, all modification treatments significantly altered the contents of the major components and the color. Regarding moisture content, all groups showed a decrease (*p* < 0.05), with the “combined modification with enzymatic hydrolysis” group having the lowest value (3.34%), indicating that modification can enhance water-holding capacity or improve drying properties [27]. Crude fat content decreased slightly after fermentation (*p* < 0.05), whereas enzymatic hydrolysis and combined treatments resulted in significant reductions (as low as 0.60%). The enzymatic modification of the starch network may lead to physical entrapment of fat, thereby reducing its extractability by organic solvents [28]. Crude protein was significantly reduced during modification (*p* < 0.05). This may be attributable to the utilization of proteins as a nitrogen source during fermentation and their loss during the subsequent modification process, resulting in a significant decrease in content [29]. In particular, the “pre-fermentation combined modification” group dropped to 5.03%, indicating that the combined treatment resulted in a higher degree of protein hydrolysis. Starch content decreased across all modification groups (*p* < 0.05), with the lowest value in the enzymatic hydrolysis group (67.51%) and a relatively higher value in the combined treatment group, which may be related to the effects of the degree of enzymatic hydrolysis and fermentation on starch structure [30].

Regarding color, after modification, lightness (L* value) decreased, while redness (a* value), yellowness (b* value), and the whiteness index (W) all increased, shifting the powder toward reddish and yellowish hues while improving whiteness. The changes were most pronounced in the single-fermentation treatment (*p* < 0.05), likely due to organic acids and enzymes produced by lactic acid bacteria that promoted the release of colored substances and led to partial disintegration of starch granules [31,32]. The effect of single enzymatic hydrolysis was more moderate, primarily altering pigment binding by removing branched starch chains [33,34]. The combined treatment yielded results intermediate between the two, indicating an interaction that stabilizes color. Overall, these modifications enhance the visual acceptability of products and functional foods, providing a basis for developing low-fat and functional highland barley products.

### 3.2. SEM Morphology Determination Results of Highland Barley Flour

**Figure 1:**
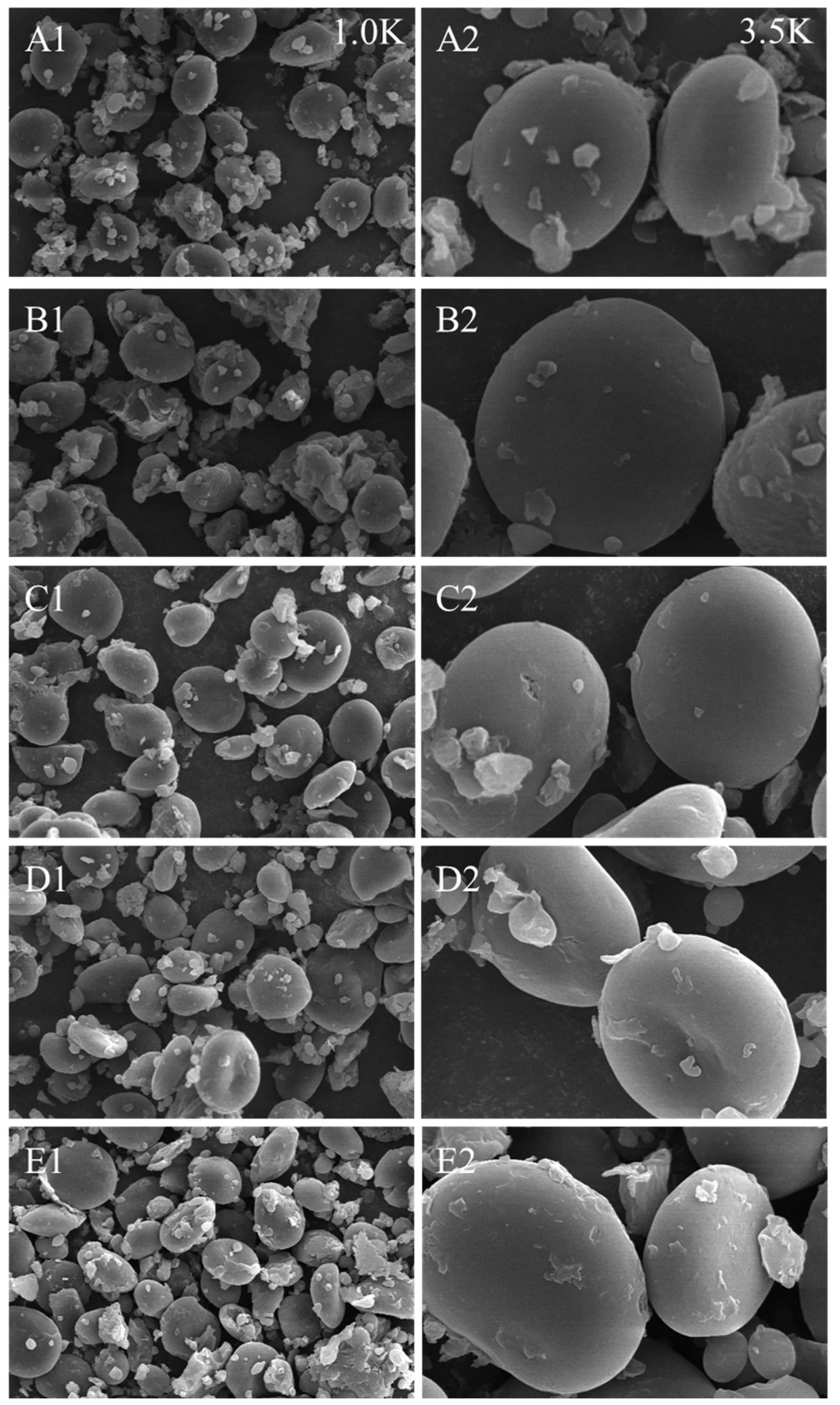
Scanning electron microscopy images of different modified highland barley flours. (**A1**,**A2**): HB, raw powder; (**B1**,**B2**): LPF, Lactobacillus plantarum fermentation; (**C1**,**C2**): PH, pullulanase hydrolysis; (**D1**,**D2**): LPF-PH, Lactobacillus plantarum fermentation followed by pullulanase hydrolysis; (**E1**,**E2**): PH-LPF, pullulanase hydrolysis followed by Lactobacillus plantarum fermentation; Left column 1.0K; Right column 3.5K.

Scanning electron microscopy (SEM) can clearly reveal the microstructure of various modified highland barley powders, facilitating in-depth research into the effects of modification treatments on their structural characteristics and functional properties. Figure 1 shows that the SEM morphology of the original powder retains structurally intact starch granules, which are primarily smooth and oval in shape. Partially dispersed non-starch components or aggregates formed on the surface are visible around the granules; granule sizes are classified as large (10–30 μm) and small (<10 μm). Following Lactobacillus plantarum fermentation, distinct pores and roughening appeared on the particle surfaces, accompanied by a looser particle structure and reduced aggregation of non-starch components on the surface. This indicates that microbial metabolism partially degraded the starch and proteins, promoting structural deconstruction [35,36]. Pullulanase hydrolysis, on the other hand, results in more pronounced erosion and an etched-like surface, but the dispersion and encapsulation of non-starch components remain largely unchanged, suggesting that the enzyme primarily acts on the α-1,6 glycosidic bonds of amylopectin [37,38]. Combined treatments resulted in more pronounced fragmentation and porosity, with a further reduction in surrounding non-starch aggregates and a sparser distribution of surface-bound components; among these, combined modification with pre-enzymatic hydrolysis produced a significantly looser structure. Pre-enzymatic hydrolysis may more readily provide substrates for microbial fermentation, thereby more effectively promoting the degradation of components and the deconstruction of particle structures.

### 3.3. Particle Size Analysis

**Figure 2:**
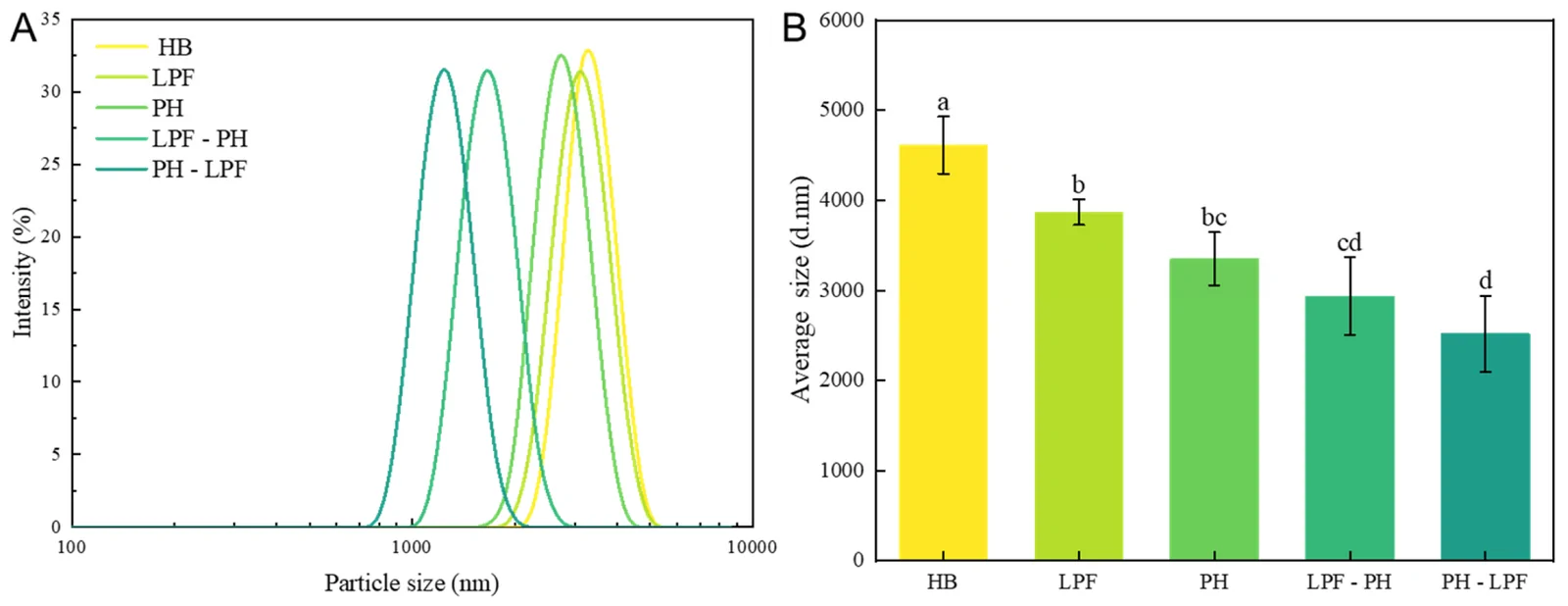
Results of particle size distribution (**A**) and average particle size measurements (**B**) for high-altitude barley flour under different modification methods; HB: raw powder, LPF: Lactobacillus plantarum fermentation, PH: pullulanase hydrolysis, LPF-PH: Lactobacillus plantarum fermentation followed by pullulanase hydrolysis, PH-LPF: pullulanase hydrolysis followed by Lactobacillus plantarum fermentation. Letters in each panel represent statistical significance (*p* < 0.05).

Determining the particle size of highland barley flour after various modifications directly affects its functional properties and processing applications. As shown in Figure 2, compared with the original powder, all samples with varying degrees of modification exhibited reduced particle size (*p* < 0.05), with peak positions shifting toward smaller particle sizes. This reduction is primarily attributed to lactic acid production during Lactobacillus plantarum fermentation and to the degradation of proteins and cell wall polysaccharides by extracellular enzymes, which promotes the release of starch granules [39]. The acidic environment may also erode particle surfaces [40]. Pullulanase hydrolysis reduces particle size by specifically hydrolyzing the α-1,6 glycosidic bonds of amylopectin, thereby disrupting particle integrity and enhancing fragmentation [41]. Among the various modification methods, the combined modification involving pre-enzymatic hydrolysis was the most significant, resulting in a particle size distribution of highland barley flour that was more concentrated in the small-particle range and exhibited a broader peak shape. This indicates that the combined modification of Lactobacillus plantarum fermentation following pullulanase hydrolysis is more effective (*p* < 0.05); this may be because fermentation weakens the matrix and enhances the enzyme’s action, and the loosened structure resulting from enzymatic hydrolysis facilitates subsequent microbial degradation [17]. Overall, the reduction in particle size enhances hydration and sensitivity to enzymatic hydrolysis, thereby promoting the release of bioactive compounds and improving processing characteristics and nutritional–functional potential.

### 3.4. Determination of Amylose and Amylopectin Content

**Figure 3:**
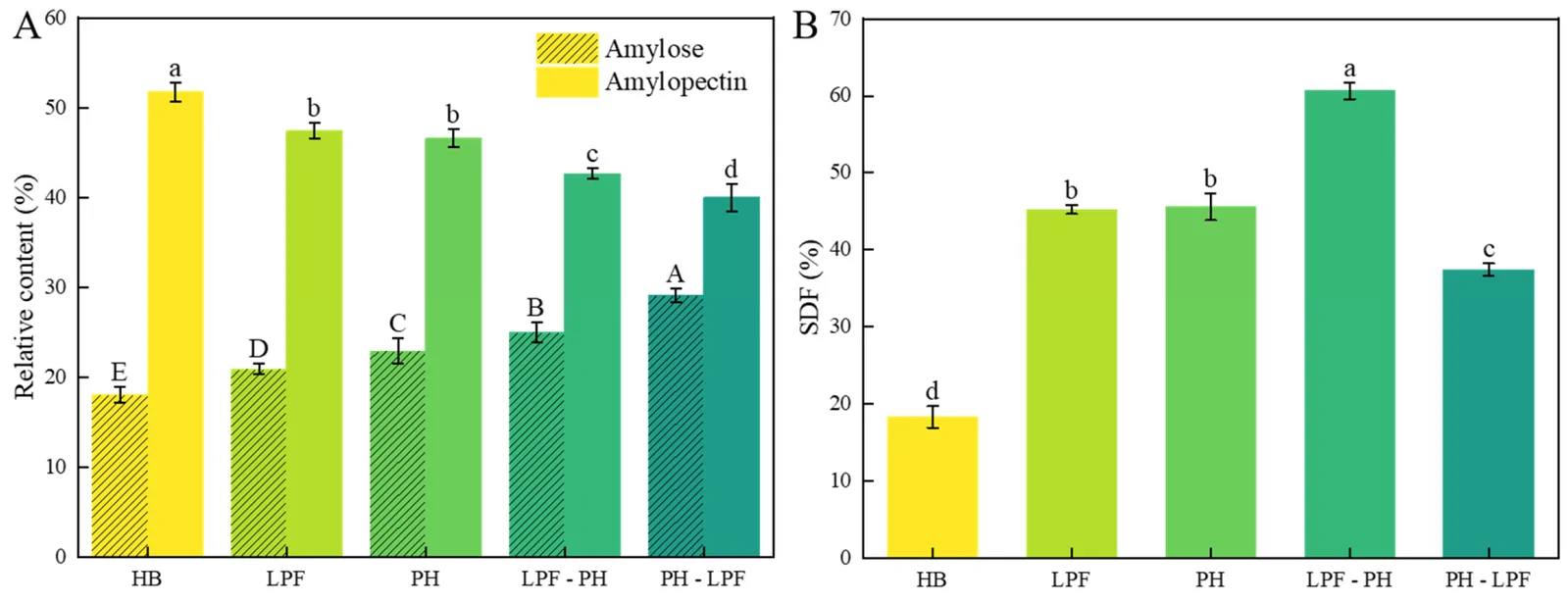
Effect of different modification methods on the amylose and amylopectin (**A**) and SDF (**B**) diffraction of highland barley flour; letters in each panel represent statistical significance (*p* < 0.05).

As shown in Figure 3A, compared with the original starch, all modification methods increased the amylose content to varying degrees and reduced the amylopectin content (*p* < 0.05). This may be because fermentation promotes the hydrolysis and rearrangement of certain macromolecular structures, resulting in an increase in amylose relative to amylopectin [42]. Among these methods, enzymatic hydrolysis specifically hydrolyzes α-1,6 glycosidic bonds, thereby more directly cleaving amylopectin chains [43,44]. Among the treatments, the “combined modification with pre-enzymatic hydrolysis” exhibited the highest proportion of linear amylose (29.09%) and the lowest proportion of branched amylose (39.99%). This indicates that, after pullulanase hydrolysis preferentially disrupts the branched structure and exposes linear fragments, the remaining branched and linear components are more readily utilized by the microbial enzyme systems during subsequent Lactobacillus plantarum fermentation [45,46]. Functionally, an increased proportion of linear starch typically promotes the formation of resistant starch, thereby reducing digestion rates and delaying the glycemic response.

### 3.5. Determination of Soluble Dietary Fiber Content

Soluble dietary fiber (SDF) is a key factor influencing the processing properties of cereal flours, alongside starch. As shown in Figure 3B, the SDF content of highland barley flour subjected to different modification treatments increased significantly (*p* < 0.05), rising from 18.32% in the original flour to 60.64%. Through the secretion of extracellular cell-wall-degrading enzymes (e.g., cellulase and xylanase) during fermentation, *Lactobacillus plantarum* depolymerizes the insoluble cellulose–hemicellulose network [47]. As a result, a substantial fraction of the insoluble dietary fiber (IDF) component is directly converted into soluble high-molecular-weight arabinoxylan, leading to an increase in the soluble dietary fiber (SDF) content [48]. Pullulanase hydrolysis specifically cleaves the α-1,6 glycosidic bonds in starch, disrupting its compact structure and releasing insoluble fiber bound to the starch [49]. However, the combined modification involving pre-fermentation yields the most significant results (*p* < 0.05). This may be because pre-fermentation creates more favorable conditions for subsequent enzymatic action, thereby producing a stronger synergistic effect. Large amounts of short-chain linear dextrins can be generated and subsequently converted into resistant dextrins and soluble oligosaccharides. The increase in SDF content enhances the gut-health-promoting effects of highland barley flour and effectively improves food utilization and development, and the modified flour can serve as a premium base ingredient for high-fiber biscuits or meal-replacement bars [50].

### 3.6. Results of Fourier Transform Infrared Spectroscopy Analysis

**Figure 4:**
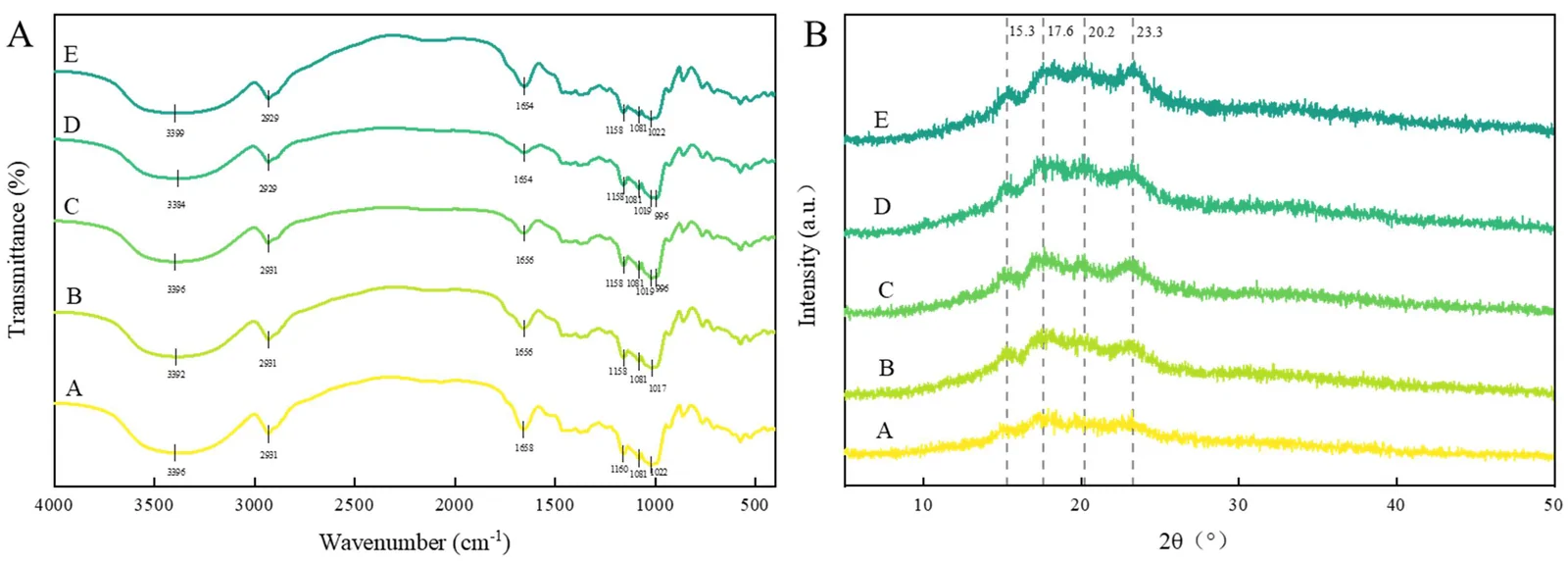
Effect of different modification methods on the FTIR (**A**) and X-ray (**B**) diffraction of highland barley flour; A: HB, raw powder; B: LPF, Lactobacillus plantarum fermentation; C: PH, pullulanase hydrolysis; D: LPF-PH, Lactobacillus plantarum fermentation followed by pullulanase hydrolysis; E: PH-LPF, pullulanase hydrolysis followed by Lactobacillus plantarum fermentation.

Fourier transform infrared spectroscopy can identify the functional groups present in a sample based on absorption peaks at different wavelengths. By analyzing the positions of these absorption peaks, it is possible to infer the functional groups and molecular bonds of major components, such as carbohydrates, and to characterize changes in the short-range-ordered structure of starch. As shown in Figure 4A, the Fourier transform infrared spectroscopy results indicate that different modifications significantly alter the molecular structure of highland barley flour. Peaks at 3400 cm^−1^ (O–H), 2930 cm^−1^ (C–H), and 1655 cm^−1^ (C=O) correspond to polysaccharide-related functional groups, respectively; the region between 1160 and 990 cm^−1^ (C–O–C) is associated with glycosidic ether bonds. Compared to the original powder, Lactobacillus plantarum fermentation (B) attenuated the polysaccharide-related peaks, while pullulanase hydrolysis (C) caused changes in peak intensity in the C–O–C region due to the hydrolysis of α-1,6 glycosidic bonds; the combined treatments (D, E) exhibited differences depending on the order of processing [51,52]. A strong O–H stretching vibration peak was observed for the native flour. Following modification, the peak underwent a shift with decreased intensity, which is indicative of the destruction of the hydrogen bonding network [53]. Analysis of starch conformation-sensitive regions (995, 1022, 1047 cm^−1^) revealed that R1047/1022 decreased while R1022/995 increased, indicating a decline in the double-helix ordered structure and short-range order [54], with the combined modification involving enzymatic hydrolysis (E) showing the most pronounced disruption. These structural changes help reduce crystallinity and enhance the material’s potential for application in functional foods.

### 3.7. X-Ray Diffraction Analysis

As shown in Figure 4B, all treatments resulted in a marked loss of the native A-type crystalline structure of the original powder, with characteristic diffraction peaks generally broadened and reduced in intensity. However, the extent of disruption varied considerably among the different modification methods. Single fermentation (B) or enzymatic hydrolysis (C) induced partial amorphization via organic acid erosion and cleavage of α-1,6-glycosidic bonds, respectively, whereas their combined modifications (D and E) exerted a synergistic disruptive effect, leading to a more pronounced transition toward an amorphous state [55]. At the same time, a new diffraction peak at 2θ = 20.2° appears, which can be attributed to the formation of starch–lipid complexes and the resulting V-type crystal structure, suggesting that the hydrogen bond network was likely weakened or partially disrupted under the applied conditions [56]. For the combined treatments, D: combined modification with fermentation first; E: combined modification with enzymatic hydrolysis first), both exhibited further broadening of the diffraction peaks and reduced intensity, suggesting that the combined treatment can more effectively degrade starch molecules and disrupt their crystalline regions. Among these, the combined modification with enzymatic hydrolysis first (E) showed the most pronounced changes in peak shape near 17.6° and 23.3°, indicating that this sequence favors the formation of more amorphous structures and/or promotes the generation of V-type starch–lipid complexes [57,58].

### 3.8. DSC Results

Differential scanning calorimetry (DSC) can characterize thermal properties related to starch processing, nutritional quality, and food functionality; its thermal behavior is associated with amylose content, chain-length distribution, and the double-helix structure in the crystalline regions of the granules [59]. As shown in Table 2, Wang et al. [60] and other studies have shown that, compared to the original starch, both the onset temperature and peak temperature exhibited distinct changes after treatment with Lactobacillus plantarum fermentation and pullulanase hydrolysis, while the enthalpy of heat absorption decreased significantly (*p* < 0.05). This indicates that microbial metabolic activity during fermentation decomposed some non-starch substances [61]. The enzymatic hydrolysis process disrupted part of the starch’s crystalline structure, thereby improving its thermal stability and reducing the energy required for gelatinization [62]. However, combined modification treatments (combined modification with prior fermentation; co-modification with pre-enzymatic hydrolysis) further reduced the enthalpy of heat absorption; in particular, the co-modification with pre-enzymatic hydrolysis exhibited the lowest enthalpy of heat absorption, suggesting a stronger effect, which may result in greater solubilization of amylose or rearrangement of the branched structure, thereby significantly reducing (*p* < 0.05) the enthalpy of gelatinization [63]. Overall, modification significantly regulates thermal processing properties by reshaping crystal order and molecular structure, providing a basis for functional optimization.

### 3.9. Determination of Water and Oil Retention

**Figure 5:**
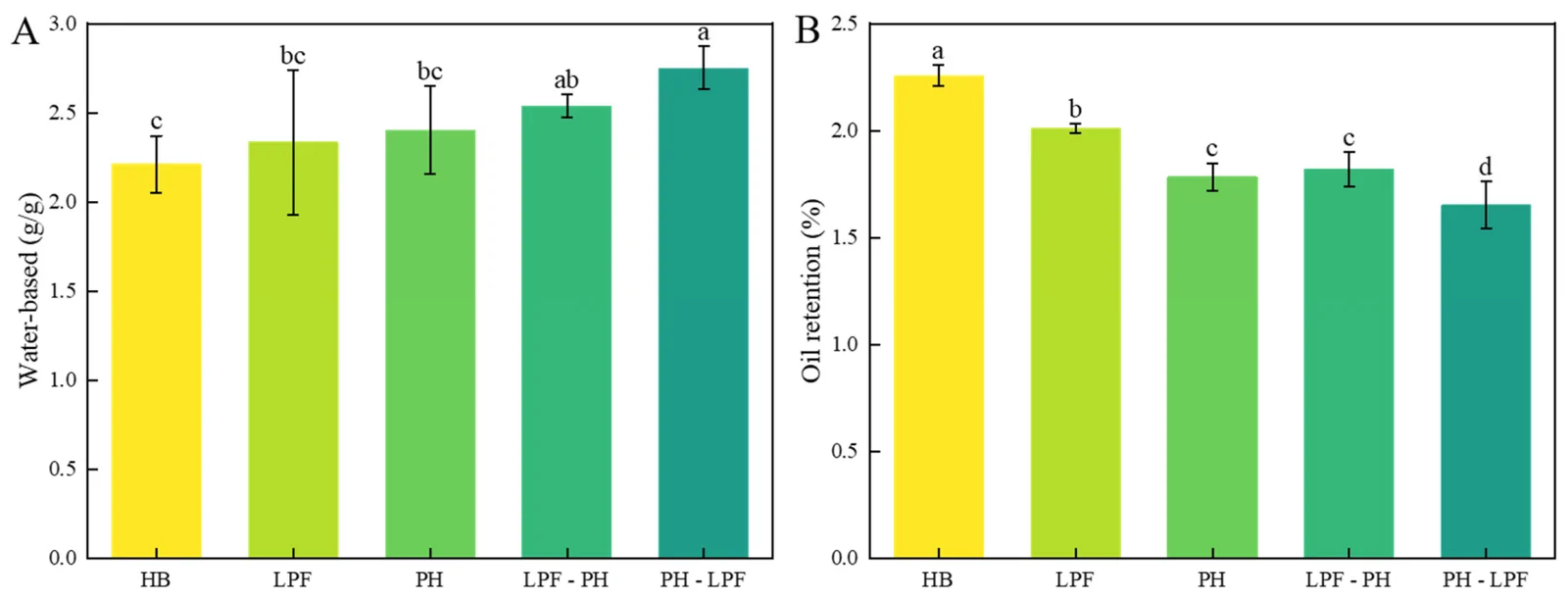
Effects of different modification methods on the water-holding capacity (**A**) and oil-holding capacity (**B**) of highland barley flour; letters in each panel represent statistical significance (*p* < 0.05).

Determining the water- and oil-holding capacities of food products hinges on precise control of their textural quality, processing efficiency, and consumer experience. As shown in Figure 5, the water-holding capacity of modified highland barley flour is higher than that of the original flour (*p* < 0.05), suggesting that the modified starch exhibited greater susceptibility to water absorption and fermentation, leading to the production of organic acids, partial hydrolysis, exposure of hydrophilic groups, and an increase in the amorphous region [64]. Pullulanase hydrolysis generates more short-chain/microporous structures, facilitating water entry and binding; in combined treatments, particularly the combined modification involving pre-enzymatic hydrolysis, structural loosening and hydrogen-bonding sites are further enhanced, resulting in the most significant improvement in water-holding capacity [65].

Compared with the oil-holding capacity of the original powder, the oil-holding capacities of all modified samples decreased (*p* < 0.05). The primary reason is that Lactobacillus plantarum fermentation destroyed the hydrophobic binding sites on the particle surface and promoted starch degradation/structural rearrangement [32,66], thereby reducing the physical space available for oil retention. Pullulanase hydrolysis reduces branching by cleaving α-1,6 glycosidic bonds and increases the proportion of linear chains; furthermore, the combined enzymatic hydrolysis in the co-treatment process further reinforces this effect, resulting in a more ordered or denser structure, which consequently leads to a further reduction in oil retention [67].

In terms of functionality, enhanced water-holding capacity helps improve texture and delay staling; reduced oil-holding capacity minimizes fat adsorption during processing, making the material more suitable for low-fat, healthy products.

### 3.10. Determination of the Effects of Textural Properties

**Figure 6:**
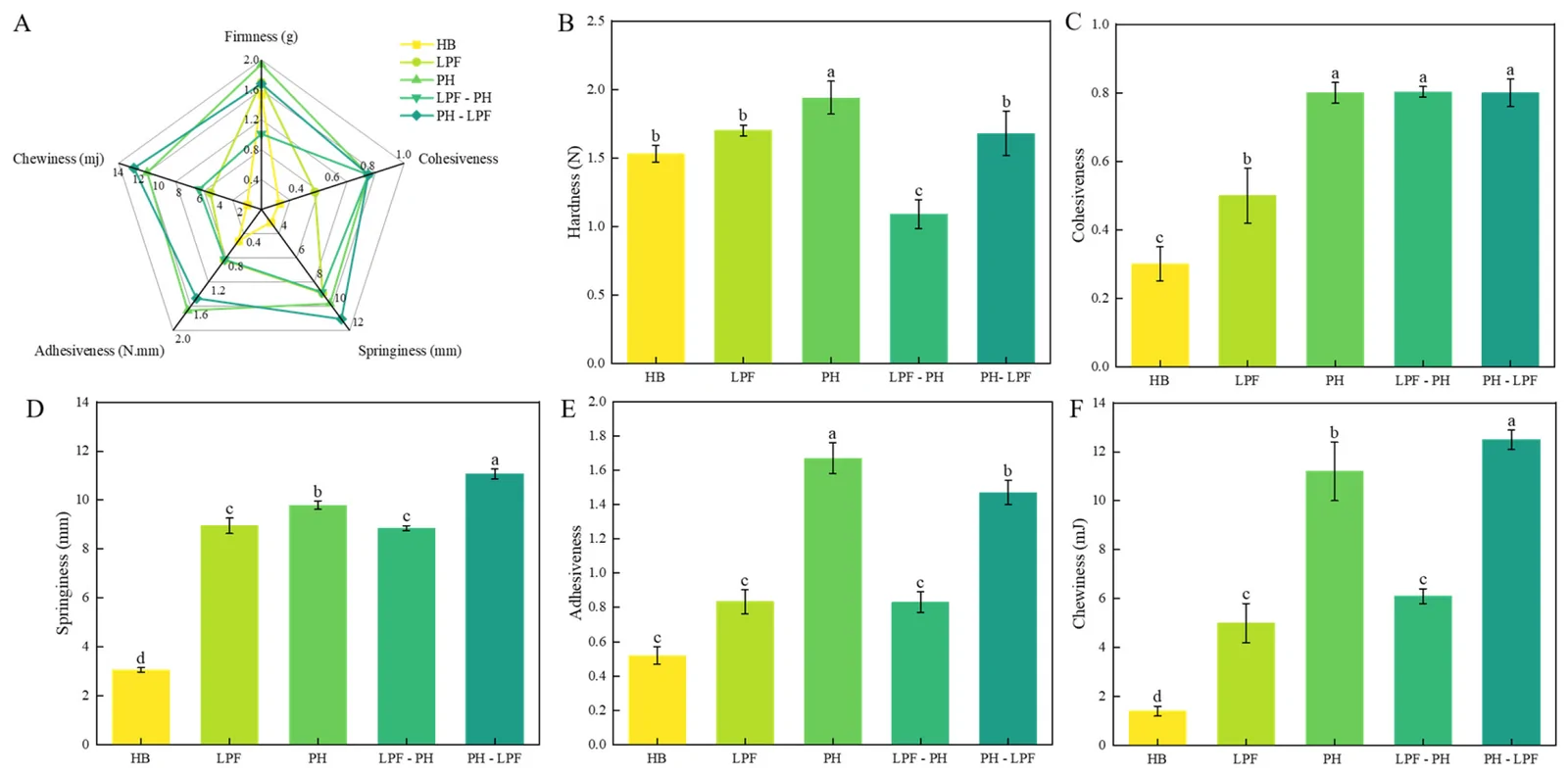
Effect of different modification methods on the texture properties of highland barley flour; (**A**): Textural characteristics radar; (**B**): Firmness; (**C**): Cohesiveness; (**D**): Springiness; (**E**): Adhesiveness; (**F**): Chewiness; letters in each panel represent statistical significance (*p* < 0.05).

As shown in Figure 6, different modifications significantly altered the texture of the highland barley dough (*p* < 0.05). The untreated flour, owing to its intact starch crystalline structure and strong intermolecular hydrogen bonds, formed a weak gel network, exhibiting lower hardness, elasticity, and chewiness. After modification by Lactobacillus plantarum fermentation, the starch underwent partial acid hydrolysis and microstructural reorganization, resulting in a slight increase in hardness and a significant improvement in cohesiveness and elasticity, suggesting the initial formation of a gel network [68,69]. Pullulanase hydrolysis results in extensive debranching, producing more short-chain linear components, which promote molecular rearrangement and strengthen the gel network. This significantly enhances hardness (*p* < 0.05), viscosity, and chewiness, forming a dense and resilient structure [70]. In the combined modification process, the overall textural parameters (hardness, chewiness) of the pre-fermented treatment were lower than those of the enzymatic hydrolysis alone, suggesting that the excessive enhancement observed with enzymatic hydrolysis was reduced upon moderate fermentation; conversely, the pre-enzymatic hydrolysis treatment achieved maximum elasticity while maintaining high hardness and chewiness (*p* < 0.05), exhibiting textural characteristics that combine both strength and resilience. Functionally, standalone enzymatic hydrolysis increases resistant starch, while fermentation improves digestibility and flavor; combined processing demonstrates greater synergistic potential in regulating digestion rate, enhancing satiety, and improving textural acceptability [14].

### 3.11. Results of Rheological Property Measurements

**Figure 7:**
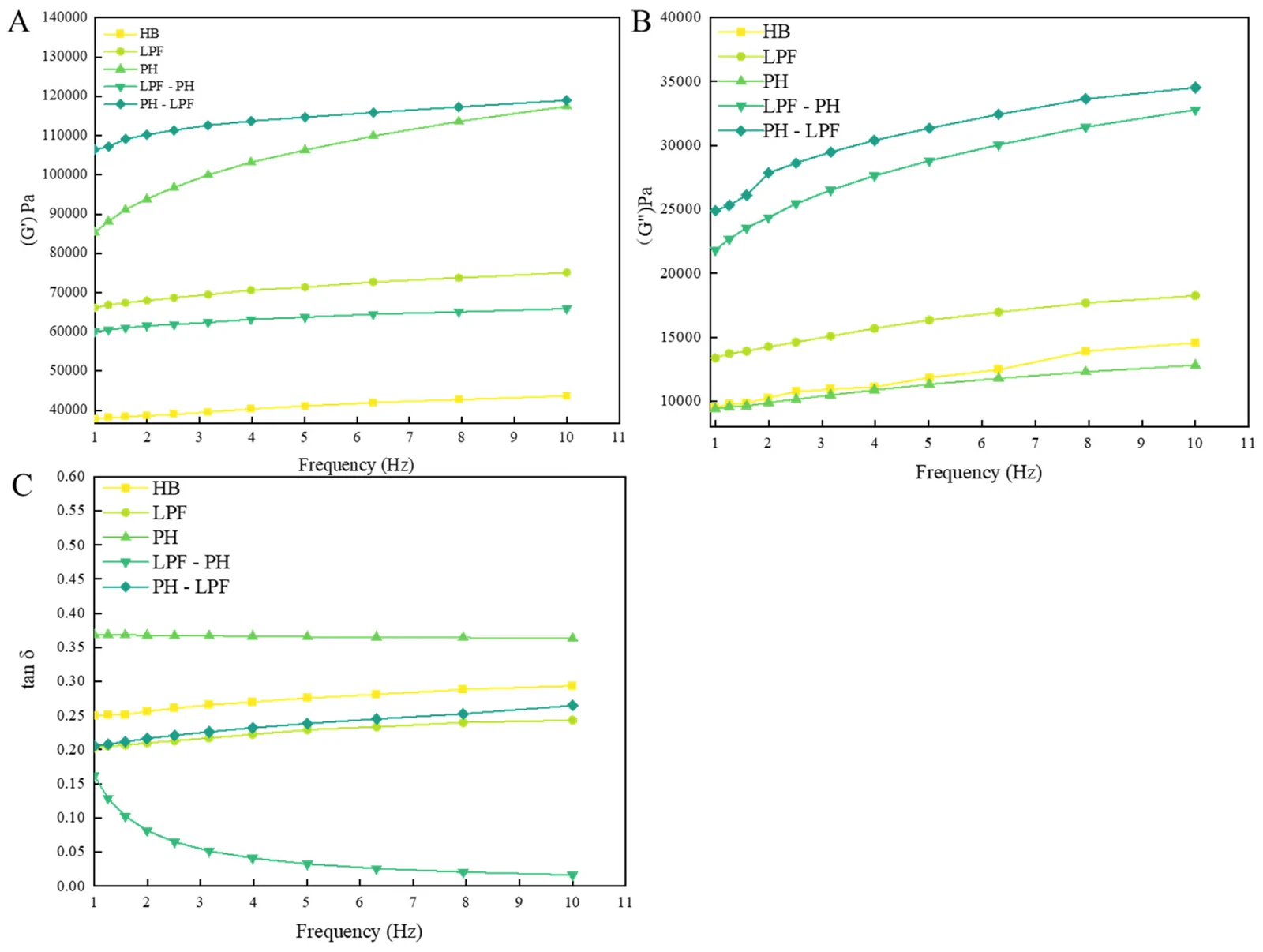
Effects of different modification methods on the elastic modulus (**A**), viscous modulus (**B**), and loss tangent (**C**) of highland barley flour dough.

By determining the rheological data of differently modified highland barley flour, we can quantitatively evaluate the effects of various modification treatments on the rheological properties of highland barley flour dough, compare the advantages and disadvantages of each modification method in terms of improving dough processing performance and workability, and thereby identify the optimal modification process. As shown in Figure 7, different modifications significantly alter the rheological properties of highland barley flour (*p* < 0.05). Compared to the original flour, Lactobacillus plantarum fermentation primarily involves microbial acid production and enzymatic partial degradation and modification of starch [69]. Although this increases the modulus, the improvement is less pronounced than that achieved by pullulanase hydrolysis. Enzymatic hydrolysis selectively breaks the α-1,6 glycosidic bonds in amylopectin, generating more linear short-chain starch, which promotes ordered interchain rearrangement and cross-linking [71]. This significantly increases the elastic modulus G’ and viscous modulus G”, while reducing the tangent of the loss angle tan δ. A stronger dough network enhances gas retention during proofing, predicting a higher specific volume and finer crumb structure in leavened products [72,73]. In the combined modification process, the modulus values for “enzymatic-hydrolysis-first combined modification” were higher than those for “fermentation-first combined modification”, suggesting that enzymatic hydrolysis first breaks down branched chains and exposes linear chains, followed by further modification through fermentation, which is conducive to the formation of a denser and more stable gel network and improves storage stability. This mechanism is also consistent with the results of the texture analysis (*p* < 0.05). Overall, the combined modification disrupts hydrogen bonds, decreases crystallinity, restructures the starch molecular network, improves processability and resistance to aging, and exhibits potential digestive stability [17]. This feature is particularly valuable for compensating the common textural drawbacks (e.g., crumbliness, poor cohesiveness, and rapid staling) in gluten-free bread, pasta, or snack matrices, offering a natural and cost-effective alternative to synthetic hydrocolloids.

## 4. Conclusions

This study systematically investigated the effects of two different sequences of combined modification (Lactobacillus plantarum fermentation, pullulanase hydrolysis) and combined modification involving pre-enzymatic hydrolysis followed by fermentation and combined modification involving pre-fermentation followed by enzymatic hydrolysis on the physicochemical and functional properties of highland barley flour. The results showed that, compared with the original flour, all modification methods significantly altered the color and basic nutritional components of the highland barley flour. The amylose content increased, the amylopectin content decreased, and the soluble dietary fiber content rose significantly. Particle size analysis and scanning electron microscopy (SEM) revealed that, after the various modification treatments, the starch and non-starch structures underwent noticeable changes. FTIR results showed hydrogen-bond disruption, increasing water accessibility and hydration. Consistently, weakened XRD diffraction peaks and the decreased enthalpy (ΔH) from DSC reflected reduced starch crystallinity and loss of ordered structure. These changes promoted more complete gelatinization and the formation of a denser, more continuous gel network, yielding a more homogeneous starch gel with enhanced mechanical strength and improved dough textural and rheological properties. Among the four modification methods, the combined enzymatic hydrolysis and water treatment yielded the most significant improvement in the properties of the highland barley flour, i.e., the combined modification using Lactobacillus plantarum fermentation and pullulanase hydrolysis. In particular, the combined treatment of pullulanase hydrolysis followed by Lactobacillus plantarum fermentation (PH-LPF) was superior to all single treatments in improving nutritional and functional properties. This may provide a more favorable structural basis for the subsequent efficient utilization of the substrate by *Lactobacillus plantarum*, possibly attributable to the initial precise cleavage of α-1,6-glycosidic bonds by pullulanase, which releases linear fragments. This further reshapes the ordered structure of starch and promotes the formation of a denser gel network. At the same time, this combined modification effectively improves the release of nutritional components, structural porosity, and related functional characteristics of highland barley flour, providing a theoretical basis and technical support for the development of functional highland barley ingredients.

## Figures and Tables

**Table 1:** Effects of Different Modifications on the Color of Highland Barley Flour (Values Expressed on a Dry Basis, Excluding Moisture).

	HB	LPF	PH	LPF-PH	PH-LPF
Moisture (%)	7.19 ± 0.02 ^a^	6.22 ± 0.03 ^b^	4.57 ± 0.20 ^c^	4.02 ± 0.10 ^d^	3.34 ± 0.10 ^a^
Crude Fat (%)	2.12 ± 0.02 ^a^	1.93 ± 0.03 ^b^	0.77 ± 0.15 ^d^	0.70 ± 0.10 ^d^	0.60 ± 0.10 ^d^
Crude Protein (%)	9.06 ± 0.10 ^a^	7.45 ± 0.30 ^b^	8.50 ± 0.25 ^a^	5.03 ± 0.10 ^c^	6.75 ± 0.71 ^b^
Starch (%)	69.81 ± 0.10 ^a^	68.33 ± 0.33 ^c^	67.51 ± 0.50 ^d^	68.66 ± 0.50 ^c^	69.08 ± 0.04 ^b^
L*	92.22 ± 0.46 ^a^	87.24 ± 0.07 ^c^	88.35 ± 0.19 ^b^	88.80 ± 0.13 ^b^	88.50 ± 0.39 ^b^
a*	1.05 ± 0.04 ^a^	2.44 ± 0.03 ^c^	2.00 ± 0.15 ^b^	1.94 ± 0.0 ^b^	2.30 ± 0.08 ^a^
b*	5.00 ± 0.24 ^d^	9.34 ± 0.08 ^a^	7.43 ± 0.29 ^c^	7.68 ± 0.03 ^c^	8.40 ± 0.13 ^b^
W	9.31 ± 0.15 ^d^	16.00 ± 0.20 ^a^	13.96 ± 0.40 ^c^	13.72 ± 0.20 ^c^	14.43 ± 0.07 ^b^

Values with different superscripts within a column indicate significant differences (*p* < 0.05). Data are represented as mean ± standard deviation (SD).

**Table 2:** DSC parameters of highland barley flour with different modification methods.

	T_o_ (°C)	T_P_ (°C)	ΔH (g/J)
HB	45.81 ± 0.34 ^d^	57.28 ± 0.06 ^c^	57.91 ± 0.06 ^a^
LPF	50.62 ± 0.09 ^a^	59.65 ± 0.18 ^a^	49.67 ± 0.22 ^b^
PH	45.89 ± 0.10 ^a^	56.97 ± 0.22 ^c^	50.30 ± 0.21 ^b^
LPF-PH	47.10 ± 0.0.1 ^c^	55.22 ± 0.05 ^d^	38.53 ± 0.11 ^c^
PH-LPF	48.70 ± 0.11 ^b^	57.97 ± 0.03 ^b^	28.89 ± 0.41 ^d^

HB: raw powder LPF: Lactobacillus plantarum fermentation; PH: pullulanase hydrolysis; LPF-PH: Lactobacillus plantarum fermentation followed by pullulanase hydrolysis; PH-LPF: pullulanase hydrolysis followed by Lactobacillus plantarum fermentation. Values with different superscripts within a column indicate significant differences (*p* < 0.05). Data are represented as mean ± standard deviation (SD).

## Data Availability

The original contributions presented in this study are included in the article. Further inquiries can be directed to the corresponding authors.
